# Prescreening with FOBT Improves Yield and Is Cost-Effective in Colorectal Screening in the Elderly

**DOI:** 10.1155/2014/179291

**Published:** 2014-04-06

**Authors:** Shashideep Singhal, Kinesh Changela, Puneet Basi, Siddharth Mathur, Sridhar Reddy, Mojdeh Momeni, Mahesh Krishnaiah, Sury Anand

**Affiliations:** ^1^Division of Gastroenterology, Department of Internal Medicine, The Brooklyn Hospital Center, Brooklyn, NY 11205, USA; ^2^Digestive and Liver Diseases, Columbia University Medical Center, 5141 Broadway, New York, NY 10034, USA

## Abstract

*Background*. Utilization of colonoscopy for routine colorectal cancer (CRC) screening in the elderly (patients over 75) is controversial. This study was designed to evaluate if using fecal occult blood test (FOBT) to select patients for colonoscopy can improve yield and be a cost- effective approach for the elderly. * Methods*. Records of 10,908 subjects who had colonoscopy during the study period were reviewed. 1496 (13.7%) were ≥75 years. In 118 of these subjects, a colonoscopy was performed to evaluate a positive FOBT. Outcomes were compared between +FOBT group (F-Group) and the asymptomatic screening group (AS-Group). The cost-effectiveness was also calculated using a median estimated standardized worldwide colonoscopy and FOBT cost (rounded to closest whole numbers) of 1000 US $ and 10 US $, respectively. * Results*. 118/1496 (7.9%) colonoscopies were performed for evaluation of +FOBT. 464/1496 (31%) colonoscopies were performed in AS-Group. In F-Group, high risk adenoma detection rate (HR-ADR) was 15.2%, and 11.9% had 1-2 tubular adenomas. In comparison, the control AS-Group had HR-ADR of 19.2% and 17.7% had 1-2 tubular adenomas. In the FOBT+ group, CRC was detected in 5.1% which was significantly higher than the AS-Group in which CRC was detected in 1.7% (*P* = 0.03). On cost-effectiveness analysis, cost per CRC detected was significantly lower, that is, 19,666 US $ in F-Group in comparison to AS-Group 58,000 US $ (*P* < 0.05). There were no significant differences in other parameters among groups. * Conclusion*. Prescreening with FOBT to select elderly for colonoscopy seems to improve the yield and can be a cost-effective CRC screening approach in this subset. The benefit in the risk benefit analysis of screening the elderly appears improved by prescreening with an inexpensive tool.

## 1. Introduction

Colorectal cancer (CRC) is a significant cause of cancer related deaths in the USA. The United States Preventative Services Task Force (USPSTF) recommends that persons aged 50 and up be screened for colorectal cancer [1]. Currently, multiple societies have recommended colonoscopy as the gold standard test for prevention and early detection of colorectal cancer. Incidence of CRC has been shown to increase with advancing age and the decision to perform colonoscopy for CRC screening in the elderly should be based on multiple factors including comorbidities, individual's risk of cancer, and risks associated with the procedure [2]. The risks involved in undergoing a colonoscopy increase with advancing age and comorbidities [3]. There is an ongoing debate on the usefulness of colonoscopy in the elderly population due to limited life expectancy and possible harmful side effects such as adverse reactions to sedatives (particularly those patients with preexisting cardiovascular and/or pulmonary disease), colon perforation/bleeding, and dehydration associated with colon prep. FOBT, on the other hand, requires no sedation or bowel prep, while the patient experiences only minimal discomfort. Screening with FOBT has been demonstrated to reduce mortality from colorectal cancer in randomized trials [4].

Few screening trials have included the elderly patient population and none used FOBT as a prescreening tool prior to colonoscopy. This study was conducted to determine if using FOBT to select patients for colonoscopy can be a valuable approach for patients over the age of 75. The study focuses on comparing the advanced adenoma detection rate (ADR), the number of tubular adenomas detected, and the rate of colorectal cancer detection in patients with positive FOBT versus a control asymptomatic group. We have further calculated the cost-effectiveness of the two CRC screening modalities in this elderly subset.

## 2. Materials and Methods

### 2.1. Study Objective

To evaluate if using fecal occult blood test (FOBT) to select patients for colonoscopy can be a valuable approach in the elderly and if prescreening with FOBT can be a cost-effective approach in the elderly.

### 2.2. Study Design

The study was a retrospective cohort study of patients undergoing colonoscopies during 2005–2009 at our institution. The study was approved by Intuitional Review Board. Colonoscopy records of the study subjects were reviewed for demographics, indication, bowel preparation, findings, and complications. The histopathology reports of the biopsies and/or polypectomies done during colonoscopy were reviewed to determine advanced adenoma rates (ADR) and colorectal cancer detection rates.

### 2.3. Inclusion Criteria

Study included adults ≥75 years of age. Asymptomatic subjects were divided into two groups: FOBT positive (F-Group) and asymptomatic screening (AS-Group).

### 2.4. Exclusion Criteria

Patients with incomplete colonoscopy records and colonoscopy done for evaluation of symptoms such as anemia, hematochezia, weight loss, abnormal imaging were excluded.

Statistical analysis was performed using SPSS statistical software for Windows version 18.

## 3. Results

Among 10,908 subjects, who had colonoscopies during this period, 1496 (13.7%) were ≥75 years. In the F-Group (*N* = 118), 61.9% were females and 38.1% were males. Racial distribution was African Americans 79.7%, Hispanics 10.2%, and Asian Americans 10.1%.

### 3.1. Colonoscopy Indications

**Figure 1:**
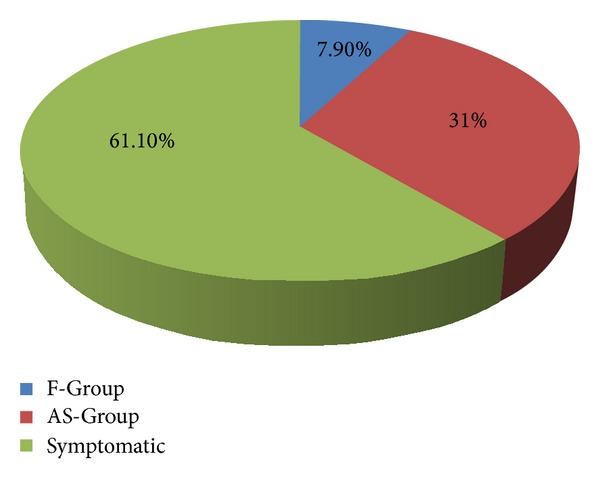
Indications of colonoscopy in subjects >75 yrs of age. F-Group (FOBT positive) and AS-Group (asymptomatic screening).

118/1496 (7.9%) colonoscopies were performed for evaluation of +FOBT. 464/1496 (31%) colonoscopies were performed in the AS-Group (see Figure 1).

### 3.2. Bowel Preparation/Completion Rates/Complications

All subjects received a standard bowel prep regimen of 1 gallon of polyethylene glycol solution followed by bisacodyl tablets. Bowel preparation during colonoscopy was good in 46.6% and suboptimal in 28%. Colonoscopy was completed up to cecum in 83.8% of subjects. No deaths or major complications requiring surgery or prolonged hospitalization were seen in the study group.

### 3.3. Colonoscopy Findings

**Figure 2:**
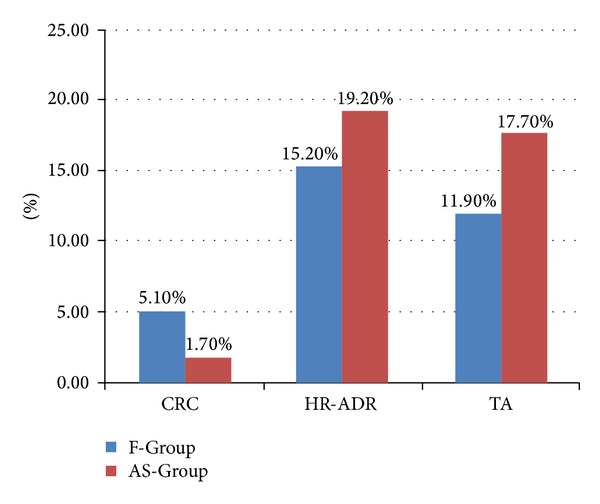
CRC detection rate (CRC), high risk adenoma detection rate (HR-ADR), and 1-2 tubular adenoma detection rate (TA) in FOBT positive (F-Group) and asymptomatic screening (AS-Group).

*Colorectal Cancer (CRC).* The CRC detection rate in the FOBT+ group was 5.1% which was significantly higher than the AS-Group in which CRC was detected in 1.7%. This was statistically significant as the *P* = 0.03 (see Figure 2).

*Adenoma Detection.* HR-ADR in the F-Group was 15.2% and 11.9% had 1-2 tubular adenomas. In comparison, the control AS-Group had HR-ADR 19.2% and 17.7% had 1-2 tubular adenomas (see Figure 2).

### 3.4. Cost-Effective Analysis

The cost-effectiveness was calculated using a median estimated standardized worldwide colonoscopy and FOBT cost (rounded to closest whole numbers) of 1000 US $ and 10 US $, respectively. Since the cost of FOBT was insignificant, the cost analysis to determine cost per CRC detected was done using the cost of colonoscopy only in F-Group and AS-Group. Cost per CRC detected was significantly lower, 19,666 US $ in F-Group (6 CRCs in 118 colonoscopies) in comparison to AS-Group 58,000 US $ (8 CRCs in 464 colonoscopies) and this was statistically significant with *P* < 0.05. There were no significant differences in other parameters among the 2 groups.

## 4. Discussion

The role of performing a colonoscopy for colorectal cancer (CRC) screening in elderly patients has been the subject of much debate. In addition, personal history of advanced adenomas or CRC, obesity, lack of physical activity, constipation, and the presence of multiple comorbidities are associated with higher adenoma and CRC detection rates during colonoscopy [5, 6]. CRC screening is still an important tool in a selected subset of subjects above age 75. In our opinion, approach for CRC screening should be individualized and the elderly with an estimated life expectancy of more than 5 years and good functional status should be offered CRC screening. The optimal modality for CRC screening has not been previously studied in this age group.

Controversy does exist regarding the safety of this procedure in the elderly as shown by prior studies. Studies have suggested that colonoscopy can be used as a screening tool in the elderly due to higher risk of neoplasia and without an increase in complications [7]. However, other studies have shown higher adverse events like infection, bleeding, and perforation after outpatient colonoscopies [8]. Other factors of concern are bowel preparation in elderly patients with multiple comorbidities.

Only half of our study subjects had a good bowel preparation during colonoscopy, while a quarter had poor bowel preparation, which is consistent with prior reports. Phosphate based preparations are shown to be associated with higher adverse event rates in elderly [9]. The factors limiting the quality of bowel preparation are likely lack of ability to follow preparation instructions adequately or presence of multiple associated comorbidities like stroke, diabetes, obesity, medications affecting motility, and limited ambulation in this subset [10, 11]. The impact of advanced age on cecal intubation rates has been shown earlier [12]. Our subset had an acceptable colonoscopy completion rate of 83% which is better than reported earlier. All the colonoscopies at our institution were done under deep sedation monitored by an anesthesiologist, which might have contributed to better completion rates.

The aim of the screening colonoscopy is early diagnosis of colonic adenomas and CRC so that appropriate intervention can be employed in early stage of diseases. The benefit of removing precancerous polyps in the elderly is limited because of life expectancy. However, detection of malignancy has a definite benefit and this will improve if an acceptable rationale for screening is adopted. Population preferences between FOBT and colonoscopy for CRC screening have been studied. FOBT was preferred as a screening test by 70.2% of the participants, colonoscopy by 9.3%, 7.4% were indecisive, and 13.1% were not interested in screening in a study of 413 randomly selected subjects [13]. A population-based, multicenter, randomized trial comparing adherence rate to screening colonoscopy compared to FOBT showed a markedly lower adherence rate in the colonoscopy group [14]. These studies show difficulties associated with implementing screening colonoscopies in the general population and are amplified in the elderly.

Our study suggests that selecting elderly subjects for colonoscopy using FOBT as a screening tool may improve the yield of finding significant pathology on colonoscopy. The benefit in the risk benefit analysis of screening the elderly appears improved by prescreening with this inexpensive tool. FOBT prescreening appears to be a cost-effective way to improve the yield of significant pathology in the elderly.
